# Prognostic utility of RECIP 1.0 with manual and AI-based segmentations in biochemically recurrent prostate cancer from [^68^Ga]Ga-PSMA-11 PET images

**DOI:** 10.1007/s00259-023-06382-2

**Published:** 2023-08-08

**Authors:** Jake Kendrick, Roslyn J Francis, Ghulam Mubashar Hassan, Pejman Rowshanfarzad, Jeremy SL Ong, Michael McCarthy, Sweeka Alexander, Martin A Ebert

**Affiliations:** 1 School of Physics, Mathematics and Computing, The University of Western Australia, Perth, Western Australia Australia; 2 Centre for Advanced Technologies in Cancer Research (CATCR), Perth, Western Australia Australia; 3 Medical School, The University of Western Australia, Crawley, Western Australia Australia; 4 Department of Nuclear Medicine, Sir Charles Gairdner Hospital, Perth, Western Australia Australia; 5 Department of Nuclear Medicine, Fiona Stanley Hospital, Murdoch, Western Australia Australia; 6 Department of Radiation Oncology, Sir Charles Gairdner Hospital, Perth, Western Australia Australia; 7 5D Clinics, Claremont, Western Australia Australia

**Keywords:** PSMA, RECIP 1.0, Artificial intelligence, Response assessment, Prostate cancer

## Abstract

**Purpose:**

This study aimed to (i) validate the Response Evaluation Criteria in PSMA (RECIP 1.0) criteria in a cohort of biochemically recurrent (BCR) prostate cancer (PCa) patients and (ii) determine if this classification could be performed fully automatically using a trained artificial intelligence (AI) model.

**Methods:**

One hundred ninety-nine patients were imaged with [^68^Ga]Ga-PSMA-11 PET/CT once at the time of biochemical recurrence and then a second time a median of 6.0 months later to assess disease progression. Standard-of-care treatments were administered to patients in the interim. Whole-body tumour volume was quantified semi-automatically (TTV_man_) in all patients and using a novel AI method (TTV_AI_) in a subset (*n* = 74, the remainder were used in the training process of the model). Patients were classified as having progressive disease (RECIP-PD), or non-progressive disease (non RECIP-PD). Association of RECIP classifications with patient overall survival (OS) was assessed using the Kaplan-Meier method with the log rank test and univariate Cox regression analysis with derivation of hazard ratios (HRs). Concordance of manual and AI response classifications was evaluated using the Cohen’s kappa statistic.

**Results:**

Twenty-six patients (26/199 = 13.1%) presented with RECIP-PD according to semi-automated delineations, which was associated with a significantly lower survival probability (log rank *p* < 0.005) and higher risk of death (HR = 3.78 (1.96–7.28), *p* < 0.005). Twelve patients (12/74 = 16.2%) presented with RECIP-PD according to AI-based segmentations, which was also associated with a significantly lower survival (log rank *p* = 0.013) and higher risk of death (HR = 3.75 (1.23–11.47), *p* = 0.02). Overall, semi-automated and AI-based RECIP classifications were in fair agreement (Cohen’s *k* = 0.31).

**Conclusion:**

RECIP 1.0 was demonstrated to be prognostic in a BCR PCa population and is robust to two different segmentation methods, including a novel AI-based method. RECIP 1.0 can be used to assess disease progression in PCa patients with less advanced disease.

This study was registered with the Australian New Zealand Clinical Trials Registry (ACTRN12615000608561) on 11 June 2015.

**Supplementary Information:**

The online version contains supplementary material available at 10.1007/s00259-023-06382-2.

## Introduction

Prostate cancer (PCa) is a commonly diagnosed malignancy that is associated with significant patient mortality [1]. If detected early, localised disease can typically be treated with radiotherapy or radical prostatectomy (RP) interventions with high success rates. However, biochemical recurrence, defined by rising serum prostate specific antigen (PSA) levels, can occur, with the possibility of the patient developing metastatic disease with a substantially poorer prognosis [2].

PCa imaging has rapidly advanced with the advent of radiotracers targeting the prostate specific membrane antigen (PSMA) transmembrane protein that is overexpressed on the majority of malignant PCa cells [3]. These PSMA-targeting radioligands can facilitate positron emission tomography/computed tomography (PET/CT) imaging with superior diagnostic performance to conventional imaging techniques, particularly for biochemically recurrent (BCR) PCa patients [4–6].

Evaluating patient response to therapeutic interventions remains critical to PCa patient care, and the quantitative analysis of medical images affords the opportunity to perform response assessments non-invasively. There exist several generalised imaging response assessment frameworks that are applied across a range of cancer types, such as the Response Evaluation Criteria in Solid Tumours (RECIST 1.1) and the PET Evaluation Response Criteria in Solid Tumours (PERCIST) [7, 8]. The updated Prostate Cancer Working Group 3 (PCWG3) criteria detail prostate cancer-specific imaging response criteria; however, they make no recommendations on PSMA imaging modalities, referring only to conventional imaging modalities such as CT and bone scintigraphy [9]. Recently, response assessment frameworks designed specifically for PSMA PET/CT images have been proposed, including the PSMA PET progression criteria (PPP) and the Response Evaluation Criteria in PSMA PET/CT (RECIP 1.0) [10, 11]. The prognostic utility of the PPP and RECIP 1.0 frameworks has been demonstrated in high disease burden metastatic castration resistant PCa (mCRPC) populations undergoing ^177^Lu-PSMA radioligand therapy, with a recent comparative study finding RECIP 1.0 to have the highest inter-reader reliability and prognostic utility in classification of progressive disease vs. non-progressive disease [12, 13]. It remains unclear; however, whether the RECIP 1.0 criteria retain its prognostic value in alternative patient populations with less advanced disease.

RECIP 1.0 requires the measurement of the change in whole-body tumour burden between baseline and follow-up imaging. Typically, this biomarker is quantified from PSMA PET scans using semi-automated techniques that require manual modifications [14, 15]. Artificial intelligence (AI) affords a unique opportunity to quantify tumour burden fully automatically, with recent work demonstrating the feasibility of using convolutional neural network (CNN) architectures to automatically segment patient disease in PSMA PET/CT scans [16–18]. AI-based disease burden quantification has the potential to facilitate fast and reproducible response assessment if integrated into frameworks such as RECIP 1.0.

The primary aim of this study was to validate the prognostic value of the radiographic RECIP 1.0 response assessment framework with respect to overall survival (OS) in a cohort of biochemically recurrent (BCR) PCa patients undergoing standard-of-care treatment. The secondary aim was to analyse whether AI-based tumour burden quantification techniques could be integrated into the RECIP 1.0 framework.

## Methods

### Patient cohort

This study included 238 patients with BCR PCa who were imaged at either Sir Charles Gairdner Hospital (SCGH) or Fiona Stanley Hospital (FSH) in Western Australia as part of a prospective trial that was registered with the Australian and New Zealand Clinical Trials Registry (ACTRN12615000608561) [4]. Inclusion criteria for the study were as follows: (i) patients must present with biochemically recurrent disease following definitive primary therapy, defined as having either a measured PSA level > 0.2ng/mL following radical prostatectomy, or a measured PSA level 2ng/mL above the nadir PSA value at 3 months following external beam radiotherapy (EBRT), and (ii) patients must have demonstrated either negative disease or oligometastatic disease (3 or less lesions) on abdominopelvic contrast CT and bone scintigraphy scans. One hundred ninety-nine of the patients recruited for this prospective study received both a baseline [^68^Ga]Ga-PSMA-11 PET/CT scan and a follow-up scan approximately 6 months later to assess disease progression—the remainder were excluded from the analysis. Therapeutic interventions for patients were administered according to standard clinical care, including any of the following: active surveillance, additional surgery, radiotherapy to the prostatic bed or metastatic lesions, and chemotherapy or androgen deprivation therapy (ADT). Ethics approval for undertaking this study was obtained from the SCGH Human Research Ethics Committee (RGS1736).

### Scan acquisition

[^68^Ga]Ga-PSMA-11 PET/CT scans were performed on either a Siemens Biograph 64 or a Siemens Biograph 128 PET/CT scanner (CTI Inc., Knoxville, TN). Patients were instructed to void their bladders prior to image acquisition. A low dose CT (50 mAs, 120 kVp) was acquired first and used for attenuation correction, with the PET emission data following immediately after with an identical field of view. Images were acquired 60 min after the intravenous injection of 2MBq/Kg of [^68^Ga]Ga-PSMA-11. PET images were reconstructed to a pixel size of 4.07 × 4.07 mm^2^, while CT images were reconstructed to a pixel size of either 0.98 × 0.98 mm^2^ or 1.52 × 1.52 mm^2^. Further details about the PET/CT scanning protocols are provided in Supplementary Table 1.

### Manual lesion delineation

Patient scans were analysed and segmented by an expert nuclear medicine physician (J.O.). Scans were interpreted according to the E-PSMA 5-point scoring criteria, where areas of increased radiotracer uptake were determined to be a lesion if they were deemed to be either ‘definitely’ or ‘probably’ positive [19]. All other sites were considered negative and excluded from the analysis. A semi-automated delineation procedure was followed; whereby, a threshold of 3 SUV_bw_ was applied to the PET image to begin with. This segmentation volume was then manually adjusted by removing any physiologic uptake that was included in the threshold, and to insert contours for lesions missed by the threshold, yielding the final scan delineation that was used to perform the RECIP classification. Delineations were performed using MIM Encore software (MIM Software Inc., Cleveland, OH, USA).

### AI-based lesion delineation

A combination of two AI models, a classification model and a segmentation model, was used to perform fully automated lesion delineation of patient scans. The classification model, which is a 3D U-Net cascade, was described in a previous study [16] and was used to determine the PSMA-positivity of patient scans. PSMA-negative scans were assigned a tumour burden of zero. PSMA-positive scans were subsequently input into a second AI model to perform fully automated segmentation of lesion sites.

The second AI model consisted of a 3D full resolution U-Net architecture trained using the nnU-Net framework [20]. The training procedure was identical to that described in [16] with the exception of the loss function which was modified to be the sum of the conventional nnU-Net loss function (dice similarity coefficient + cross entropy) and the TopK10 loss [21]. This combined loss function was chosen to force the network to focus on voxels that were difficult to identify during the training process and improve voxel-level segmentation results relative to the network reported in [16]. This segmentation model was trained on an NVIDIA GeForce RTX 3090.

### RECIP classification

**Fig. 1:**
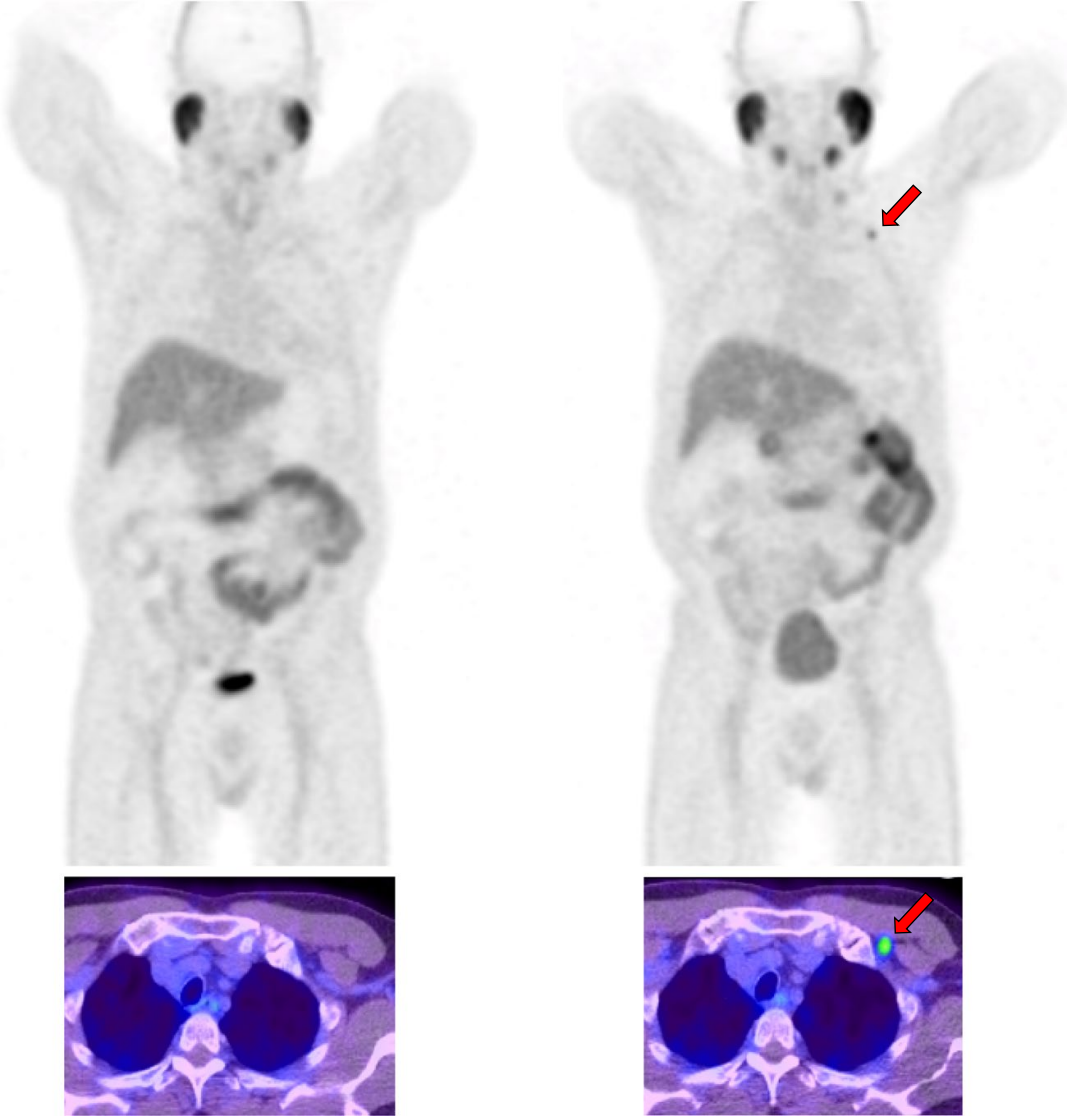
Maximum intensity projection PET images for a single patient who demonstrated RECIP progressive disease between baseline scanning (pictured left) and follow-up scan (pictured right). Patient presented with a 5.74 mL increase in tumour volume between baseline and follow-up (baseline TTV = 7.86 mL, follow-up TTV = 13.60 mL, ΔTTV (%) = 73.0%). A new nodal lesion was visible on the follow-up scan in the left supra-clavicular region (red arrows, shown above)

Whole-body total tumour volume (TTV) was calculated in the same way for both the manual (TTV_man_) and automated (TTV_AI_) segmentation methods—by summing the number of identified positive voxels and multiplying by the voxel volume. The percentage change between baseline and follow-up PSMA scans was quantified (ΔTTV). Both the manual and AI-segmented scans were retrospectively analysed to check for the presence of new lesions between baseline and follow-up. The presence of new lesions and the percentage change in new lesions were integrated into the RECIP 1.0 classification system to classify patients with progressive disease (RECIP-PD) or non-progressive disease (non RECIP-PD). The RECIP 1.0 criteria is outlined in Table 1 [11], and an example of a RECIP-PD patient is presented in Fig. 1.

**Table 1:** RECIP 1.0 response assessment definitions

Progression criteria	RECIP 1.0
PD	> 20% tumour burden increase and appearance of ≥ 1 new lesion
SD	≥ 20% tumour burden increase with no new lesions or ≥ 1 new lesion with tumour burden decline of ≥ 30% or tumour burden change between −30% and 20%
PR	Tumour burden decline of > 30% and no new lesions
CR	No lesions identified on follow-up PET

*RECIP* Response Evaluation Criteria in PSMA PET/CT, *PD* progressive disease, *SD* stable disease, *PR* partial response, *CR* complete response

### Statistical analysis

Association of RECIP classifications with patient OS was assessed using the Kaplan-Meier method with the log rank test and univariate Cox regression analysis with derivation of hazard ratios (HRs) and Harrell’s concordance index (C-index). Survival analysis was performed with the Lifelines package version 0.27.1. Concordance between the AI and manual classifications was assessed using the Cohen’s Kappa statistic (*k*) in SciPy version 1.8.0, with agreement interpreted as follows: none to slight (*k* **≤** 0.20), fair (0.20 < *k* **≤** 0.40), moderate (0.40 < *k* **≤** 0.60), substantial (0.60 < *k* **≤** 0.80), and almost perfect (0.80 < *k* **≤** 1.00) [22]. Spearman correlation coefficient was used to assess correlation between TTV_AI_ and TTV_man_ for both baseline and follow-up scans using SciPy version 1.8.0. In all cases, *p* < 0.05 was considered to be a statistically significant difference. All statistical analysis was conducted in Python version 3.9.

## Results

The median time between baseline and follow-up imaging for the cohort was 6.0 months (range: 3.2–8.8). Of the total 199 patients included for analysis, 125 were used for training of the AI model, and thus had to be excluded from AI-based automatic lesion delineation so that an unbiased estimate of AI model performance was achieved. Seventy-four patients in total therefore underwent both semi-automated and AI-based delineation. All patients were followed up from the time of follow-up scanning until either death or date of censoring to facilitate survival analysis, with a median follow-up time of 66.7 months (range: 4.7–75.4). PSMA baseline scan interpretation and other patient clinical and laboratory data were used to inform patient treatment decisions, which were made at the discretion of the treating physician. Detailed patient characteristics are summarised in Table 2. Between baseline and follow-up scanning, 89 patients (44.7%) received systemic ADT treatment, 71 (35.7%) underwent disease surveillance, 61 (30.7%) received a radiotherapy procedure, and 6 (3.0%) received chemotherapy.

**Table 2:** Patient characteristics

Characteristic	All patients (*n* = 199)	AI-tested subset (*n* = 74)
Age (y)	70 (46–90)	70 (46–83)
PSA (ng/mL)	2.70 (0.20–79.46)	1.79 (0.20–22.04)
Gleason score*		
< 8	113 (57.9%)	45 (62.5%)
≥ 8	82 (42.1%)	27 (37.5%)
Time between baseline and follow-up scan (months)	6.0 (3.2–8.8)	6.2 (5.3–8.8)
Previous definitive treatment		
Prostatectomy	123 (61.8%)	53 (71.6%)
Radiotherapy	76 (38.2%)	21 (28.3%)
Administered treatments between imaging		
Active surveillance	71 (35.7%)	29 (39.2%)
ADT	89 (44.7%)	26 (35.1%)
Radiotherapy	61 (30.7%)	23 (31.1%)
Chemotherapy	6 (3.0%)	3 (4.1%)

Continuous data is presented as the median with the range in parentheses, while nominal data is presented as the number with percentage of the whole in parentheses

^*^Data missing for 4 patients (2 in AI-tested subset)

### Manual RECIP classification

**Fig. 2:**
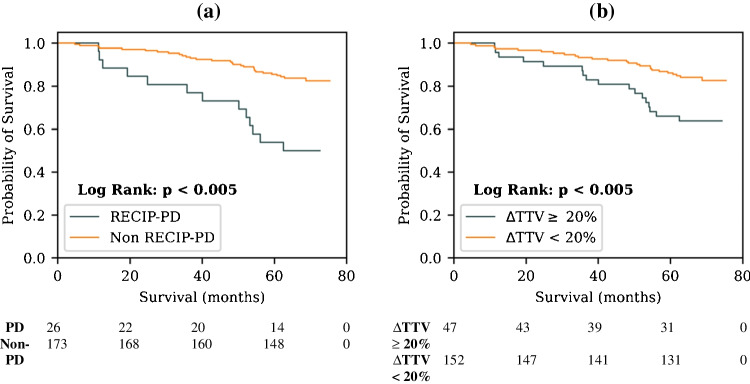
Kaplan-Meier plots showing univariate association of the categorical variables (**a)** RECIP-PD and (**b)** ΔTTV ≥ 20% with overall survival. Classifications were performed using manual tumour burden delineations. The number of patients that are still at risk at a given time point, defined as those patients that have either not experienced death or been censored, are shown below each plot (time points in the table align with the *x*-axes of the plots)

**Fig. 3:**
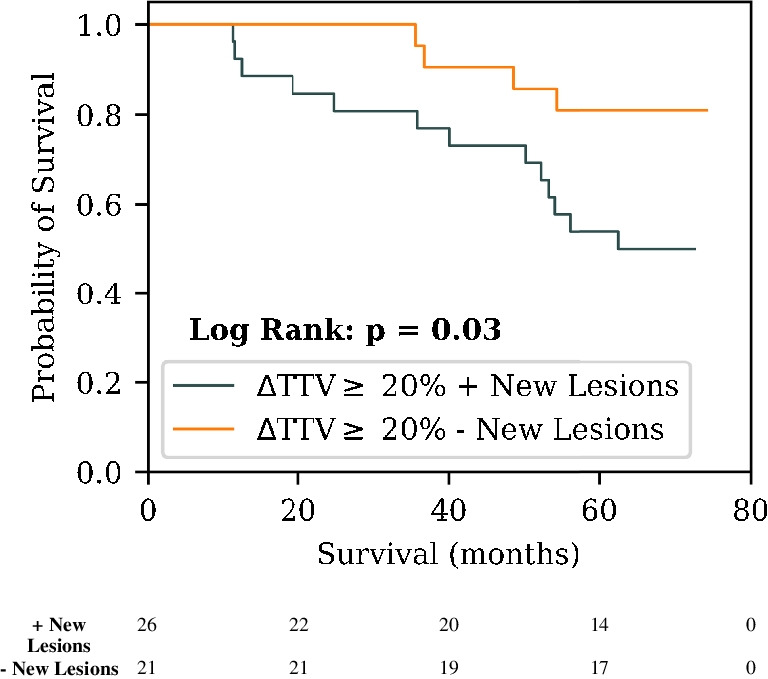
Kaplan-Meier plot showing the prognostic utility of the presence of new lesions in patients that demonstrated a ≥ 20% TTV increase from baseline imaging according to manual lesion delineations. The number of patients that are still at risk at a given time point, defined as those patients that have either not experienced death or been censored, are shown below each plot

Of the 199 patients who underwent semi-automated lesion delineation, 23.6% (*n* = 47) had a ΔTTV_man_ of greater than or equal to 20% between baseline and follow-up. Among these 47 patients, 26 also presented with new lesions (26/47 = 55.3%). Twenty-six out of the total 199 (13.1%) were therefore classified as having PD according to RECIP 1.0. Kaplan-Meier analysis reveals a statistically significant reduction in OS for RECIP-PD patients (median OS = 62.5 months) relative to non-PD patients (median OS not reached, *p* < 0.005; Fig. 2a), who also had a significantly higher risk of death (HR = 3.78 (1.96–7.28), *p* < 0.005). Patients that had just a 20% or greater ΔTTV_man_ increase also had a statistically significant lower survival probability (median OS not reached for both groups, Kaplan-Meier log rank *p* < 0.005, Fig. 2b) and higher risk of death (HR = 2.50 (1.35–4.63), *p* < 0.005) relative to those that did not. In the subset of patients with > 20% ΔTTV_man_ increase, stratified based on the presence of new lesions between baseline and follow-up, Kaplan-Meier analysis demonstrates that new lesions are associated with a significantly lower survival probability (median OS = 62.5 months vs. not reached, *p* = 0.03, Fig. 3). Cox regression analysis shows that new lesions are also associated with higher risk of death (HR = 3.22 (1.05–9.89), *p* = 0.04), confirming the hypothesis made in the original RECIP 1.0 paper in our cohort [11]. A ΔTTV_man_ greater than zero, showing increased disease burden between baseline and follow-up, was also associated with an increased risk of death (HR = 2.33 (1.27–4.28), *p* = 0.01). The prognostic utility of various ΔTTV_man_ threshold cut-off values are presented in Table 3.

**Table 3:** Prognostic value of different ΔTTV_man_ threshold cut-off values between baseline and follow-up imaging

Threshold cutoff	HR (95% CI)	*p*-value	C-index
Any TTV increase	2.33 (1.27–4.28)	0.01	0.60
10%	2.26 (1.22–4.18)	0.01	0.59
20%	2.5 (1.35–4.63)	< 0.005	0.60
30%	2.15 (1.14–4.04)	0.02	0.58
40%	2.06 (1.08–3.91)	0.03	0.57
50%	2.38 (1.25–4.52)	0.01	0.58

### AI RECIP classification

**Fig. 4:**
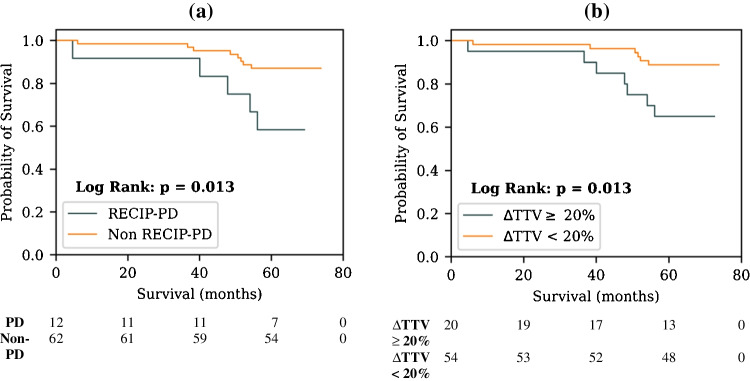
Kaplan-Meier plots showing univariate association of the categorical variables (**a)** RECIP-PD and (**b)** ΔTTV ≥ 20% with overall survival. Classifications were performed using the AI model–automated delineations. The number of patients that are still at risk at a given time point, defined as those patients that have either not experienced death or been censored, are shown below each plot (time points in the table align with the *x*-axes of the plots)

Of the 74 patients who underwent fully automated AI-based lesion segmentation, 27.0% (*n* = 20) had a ΔTTV_AI_ of greater than or equal to 20% between baseline and follow-up. Twelve of these patients also developed new disease sites (12/20 = 60%), meaning that 12 patients out of 74 (16.2%) were classified as having RECIP-PD. Kaplan-Meier analysis demonstrates a statistically significant reduction in survival probability for RECIP-PD patients relative to those without RECIP-PD (median OS not reached for both groups, *p* = 0.013, Fig. 4a), and Cox regression shows a higher relative risk of death (HR = 3.75 (1.23–11.47), *p* = 0.02). A greater than 20% ΔTTV_AI_ increase between baseline and follow-up was also associated with a significant reduction in OS (median OS not reached for both groups, Kaplan-Meier log rank *p* = 0.013, Fig. 4b) and higher risk of death (HR = 3.65 (1.23–10.89), *p* = 0.02). A ΔTTV_AI_ of more than zero was also associated with an increased risk of death (HR = 3.13 (1.05–9.32), *p* = 0.04). The prognostic value of multiple different ΔTTV_AI_ threshold cut-off values are presented in Table 4.

**Table 4:** Prognostic value of different ΔTTV_AI_ threshold cut-off values between baseline and follow-up imaging. Cohen’s *k* is also presented showing concordance between AI and manual classifications at each threshold

Threshold cutoff	HR (95% CI)	*p*-value	C-index	*k*
Any TTV increase	3.13 (1.05–9.32)	0.04	0.64	0.60
10%	3.38 (1.13–10.06)	0.03	0.64	0.58
20%	3.65 (1.23–10.89)	0.02	0.65	0.60
30%	3.97 (1.33–11.83)	0.01	0.66	0.59
40%	3.97 (1.33–11.83)	0.01	0.66	0.62
50%	4.33 (1.45–12.90)	0.01	0.67	0.59

### Concordance between AI and manual RECIP

**Fig. 5:**
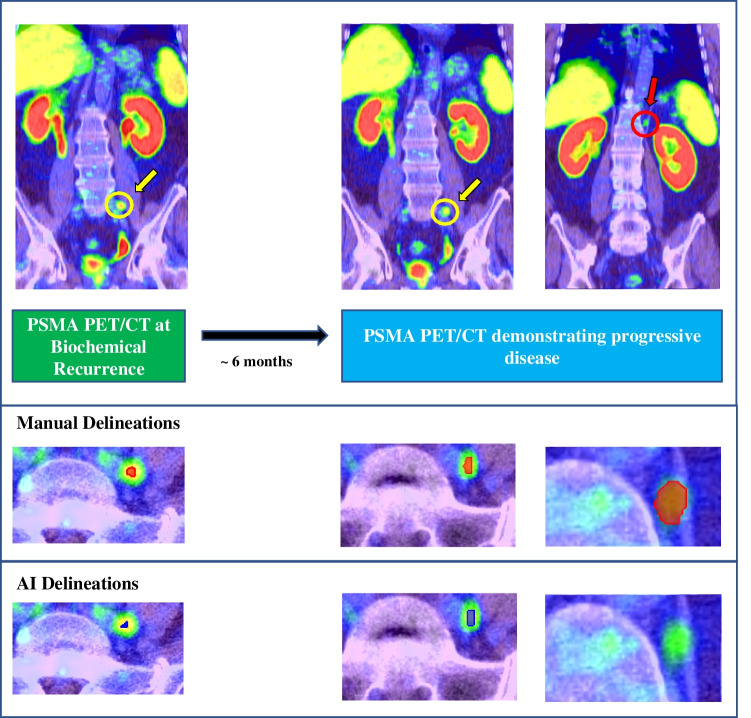
Case example of a patient demonstrating RECIP-PD according to manual interpretation, but not according to the AI model. The top row shows coronal slices of identified disease sites, by manual interpretation, at baseline and follow-up imaging. Manual and AI delineations of those disease sites in axial slices are provided directly underneath in red and dark blue, respectively. This patient (male, 68 years old, Gleason score = 9, PSA at referral = 0.23 ng/mL) presented with a single lesion in the left iliac node (yellow circle and arrow, SUV_max_ = 14.1) at baseline imaging which was successfully detected by the AI model. This lesion was also identified on PSMA PET/CT imaging 6 months later (yellow circle and arrow, SUV_max_ = 9.9) by both manual and AI scan interpretation; however, the patient also developed a new nodal disease site above the diaphragm which was only identified by human interpretation and not by the AI model (red circle and arrow). This false negative by the AI model, perhaps caused by the overall lower uptake of this lesion (SUV_max_ = 2.8), led to the discordance in RECIP classifications for this patient (RECIP-PD for manual, non RECIP-PD for AI). Despite the discordance in RECIP classification, there was concordance on whether the patient had a ΔTTV ≥ 20% between initial and follow-up imaging (ΔTTV_man_ = 325%, ΔTTV_AI_ = 54%)

The AI model and observer RECIP classifications were in agreement for 62 out of the total 74 cases (83.8%). Overall, the AI model was more in agreement with manual interpretation in non RECIP-PD cases (58/66 = 87.9%) than in RECIP-PD cases (4/8 = 50%). A confusion matrix of the RECIP classifications between AI and manual observer is presented in Table 5, and an exemplar failure case of the AI model to predict RECIP-PD is demonstrated in Fig. 5. AI RECIP classifications were overall in fair agreement with the manual interpretations (*k* = 0.31); however, much better agreement was achieved for classifying patients at various ΔTTV cut-off thresholds (moderate—substantial agreement, *k* range = 0.59–0.62; Table 4). A strong positive correlation between the TTV_AI_ and TTV_man_ measurements was found for both baseline (*r*_spearman_ = 0.94, *p* < 0.005) and follow-up scans (*r*_spearman_ = 0.88, *p* < 0.005).

**Table 5:** Confusion matrix demonstrating similarities and differences in RECIP classifications between AI model and manual interpretation

		AI RECIP	
		Non-PD	PD
Manual RECIP	Non-PD	58	8
	PD	4	4

## Discussion

Evaluating disease progression in molecular imaging is a critical component of patient care. Response assessment frameworks that are intended for clinical use should demonstrate prognostic utility in the cohort that they are utilised in. The RECIP 1.0 criteria has demonstrated its prognostic power in high disease burden mCRPC populations undergoing ^177^Lu-PSMA radioligand therapy [12, 13], but its prognostic utility in less advanced disease populations remained to be validated. In this study, we demonstrated that in a less advanced disease BCR PCa population undergoing standard-of-care treatment with a long follow-up time, the RECIP criteria retains its prognostic significance. Furthermore, we showed the feasibility of incorporating automated AI-based lesion delineations into the RECIP framework without loss of prognostic value. With the potential for AI tumour burden quantification to facilitate both fast and completely reproducible response assessment, the clinical implications of this are significant.

AI-based lesion segmentation in PSMA images is rapidly advancing, with numerous studies demonstrating the potential for fully automatic PCa lesion delineation [16–18]. To our knowledge, this work is the first to report the prognostic value of a fully automatic AI-based methodology for tumour burden quantification in a response assessment setting in prostate cancer, with previous work in this space employing semi-automated segmentation techniques. Kind et al. [12] retrospectively analysed the prognostic value of the RECIP framework in mCRPC patients undergoing ^177^Lu-PSMA radioligand therapy, with tumour burden quantified semi-automatically using the methodology developed by Seifert et al. [15]. Their results demonstrated a significantly increased risk of death for RECIP-PD patients (HR 2.69 (1.42–5.11), *p* = 0.002), a finding that was replicated in our less advanced disease population for both semi-automated (HR = 3.78 (1.96–7.28), *p* < 0.005) and AI-based (HR = 3.75 (1.23–11.47), *p* = 0.02) segmentation methods. Gafita et al. [13] in their recent comparative study utilised the semi-automated qPSMA software [14] for tumour volume quantification, yielding also a significant increased risk of death for RECIP-PD patients undergoing ^177^Lu-PSMA radioligand therapy (HR = 4.33 (2.80–6.70), *p* < 0.001) that is again similar to our results. Our novel AI-based method has the advantages of both complete reproducibility and requiring no manual modifications of the segmentation mask relative to these semi-automated techniques.

In the original RECIP 1.0 study, it was hypothesised that in patients who demonstrated a ΔTTV increase of > 20%, those who also had new lesions develop between scans would have a significantly worse survival probability relative to those who did not have new lesions [11]. Our study confirmed this hypothesis (HR = 3.22 (1.05–9.89), *p* = 0.04), suggesting that the decision to incorporate the presence of new lesions into the RECIP framework for defining RECIP-PD was valid and translates also to lower disease burden PCa populations. This analysis was done only for the semi-automated segmentation method because the sample size of patients who had AI lesion segmentation and a ΔTTV_AI_ of > 20% was small (*n* = 20).

It is noteworthy that there was higher concordance between AI and manual scan interpretation for ΔTTV > 20% (moderate agreement, *k* = 0.60) than for RECIP-PD classification (fair agreement, *k* = 0.31). The example presented in Fig. 5 demonstrates why this might be the case. This patient presented with a new nodal lesion between scans that was not detected by the AI model. This resulted in a discordant RECIP-PD classification. However, despite this false negative, both segmentation methods were in agreement about whether there was a ΔTTV > 20% (ΔTTV_man_ = 325%, ΔTTV_AI_ = 54%), because the AI model predicted a large increase in the volume of another nodal lesion in the left iliac between scans. Therefore, the incorporation of the criteria for new lesions into the RECIP framework may make it more difficult for agreement to be reached between segmentation methods, since a single false negative or positive can impact the classification. Despite this lower agreement, however, both segmentation methods demonstrated significant prognostic value in RECIP-PD classifications.

Summary assessments of disease progression at the patient level may obscure lesion-level response heterogeneity. Individual metastatic disease sites may present with underlying molecular heterogeneity which can lead to a ‘mixed response’ scenario; whereby, some lesions may respond well to treatment and reduce in volume or uptake, while others can increase in size or uptake, or new disease sites can appear within the patient [23, 24]. Published test-retest repeatability limits for metastatic PCa lesions in [^68^Ga]Ga-PSMA-11 PET images can be used to inform a lesion-level response analysis which puts the patient-level RECIP classification into further context [25]. This lesion-level response assessment analysis, which was out of the scope of the present study, is something that future work should investigate.

This study does have some limitations that should be noted. Patients were treated according to standard-of-care at the discretion of the treating physician and the patient. This means that heterogeneous treatments were administered to patients between scans, which has the benefit of being highly reflective of the treatment scenarios likely to occur in everyday clinical practice for this patient population. However, this does make it difficult to make robust conclusions about individual treatment methods on their own, and future prospective studies are necessary to elucidate the prognostic value of RECIP for specific treatment interventions in BCR PCa populations. Additionally, the segmentations generated by the AI model were used without modification or expert quality assurance. While this provides a good estimate of how well the model is performing, this is highly unlikely to be how the model is used in actual clinical practice, where AI-generated delineations will likely serve either as an initial best approximation with subsequent human modifications, or as a quality assurance check on human-generated segmentations. With such checks and balances in place, false negatives (and false positives) such as described above can potentially be mitigated. Further prospective clinical studies are required in order to quantify AI model prognostic significance when incorporated into RECIP 1.0 in a real-world clinical context [26].

## Conclusion

In this study, the prognostic value of the RECIP 1.0 criteria was demonstrated in a cohort of BCR PCa patients undergoing standard-of-care treatments. RECIP 1.0 was shown to be prognostic with two different segmentation methods—a semi-automated approach requiring manual intervention, and a fully automated AI-based method that requires no manual modifications and is completely reproducible. RECIP-PD patients classified according to both methods had a significantly higher risk of death relative to non RECIP-PD patients. Further prospective studies are required to elucidate the prognostic potential of RECIP 1.0 for specific treatment modalities in similar less advanced disease populations.

### Supplementary Information

Below is the link to the electronic supplementary material.

Supplementary file1 (PDF 92 KB)

## Data Availability

The datasets analyzed during the current study are not publicly available due to patient privacy concerns.
