# Supplementary material for: Prognostic utility of RECIP 1.0 with manual and AI-based segmentations in biochemically recurrent prostate cancer from [68Ga]Ga-PSMA-11 PET images
Source: Eur J Nucl Med Mol Imaging. 2023 Aug 8;50(13):4077–86. doi: 10.1007/s00259-023-06382-2 (PMC10611879; doi:10.1007/s00259-023-06382-2)
Supplement: Supplementary file 1 — Supplementary file1 (PDF 92 KB) [file 259_2023_6382_MOESM1_ESM.pdf]

Supplementary Table 1: Summary of the PET scanner parameters for each institution at which patients were imaged.

No. Patients	Imaging Centre	Scanner	Reconstruction Method	Pixel Spacing (mm^2)
176	SCGH	Siemens Biograph 64	PSF + Time of Flight (2i 21s)	4.07×4.07
23	FSH	Siemens Biograph 128	OSEM + Time of Flight (2i 21s)	4.07×4.07
